# Numerical Simulation of Solids Conveying in Grooved Feed Sections of Single Screw Extruders

**DOI:** 10.3390/polym14020256

**Published:** 2022-01-08

**Authors:** Florian Brüning, Volker Schöppner

**Affiliations:** Kunststofftechnik Paderborn (KTP), Paderborn University, 33098 Paderborn, Germany; volker.schoeppner@ktp.uni-paderborn.de

**Keywords:** extrusion, simulation, discrete element method, solids conveying

## Abstract

For plastic processing extruders with grooved feed sections, the design of the feed section by means of analytical calculation models can be useful to reduce experimental costs. However, these models include assumptions and simplifications that can significantly decrease the prediction accuracy of the throughput due to complex flow behavior. In this paper, the accuracy of analytical modeling for calculating the throughput in a grooved barrel extruder is verified based on a statistical design of experiments. A special focus is placed on the assumptions made in the analytics of a backpressure-independent throughput, the assumption of a block flow and the differentiation of the solids conveying into different conveying cases. Simulative throughput tests with numerical simulation software using the discrete element method, as well as experimental throughput tests, serve as a benchmark. Overall, the analytical modeling already shows a very good calculation accuracy. Nevertheless, there are some outliers that lead to larger deviations in the throughput. The model predominantly overestimates the throughputs, whereby the origin of these deviations is often in the conveying angle calculation. Therefore, a regression-based correction factor for calculating the conveying angle is developed and implemented.

## 1. Introduction

In terms of quantity, single-screw extrusion is one of the most important processing methods for thermoplastics. The screw geometry has a great influence on the throughput of the extrusion line as well as on the melt quality and is, therefore, crucial for economical operation. In contrast to smooth barrel extruders, for grooved barrel extruders, it is generally recognized that the throughput is determined by the feed section. To save costs, there are different analytical calculation methods that take solids conveying mechanisms into account so that no time-consuming trial-and-error experiments have to be conducted. There are many assumptions and simplifications that have to be made to obtain an analytical solution; for example, the assumption of the polymer pellets forming a solid bed that flows with a uniform velocity. Hence, numerical simulations using the discrete element method (DEM) are becoming more widespread for describing solids conveying in extruders because, here, relative movements between the particles are possible per definition. In this paper, a DEM simulation model is used for a virtual design of experiments (DoE) so that a large data base for evaluating existing calculation methods is obtained. Furthermore, an experimental validation is conducted with a special solids conveying test bench. For a better understanding and discussion of the topic, some general theoretical basis on solids conveying in single-screw extruders is given. This is followed by a more detailed description of the used mathematics and methods in Section 2.

### 1.1. State of the Art and Historical Development of Treatment of Solids Conveying in Feed Sections of Single-Screw Extruders

**Figure 1:**
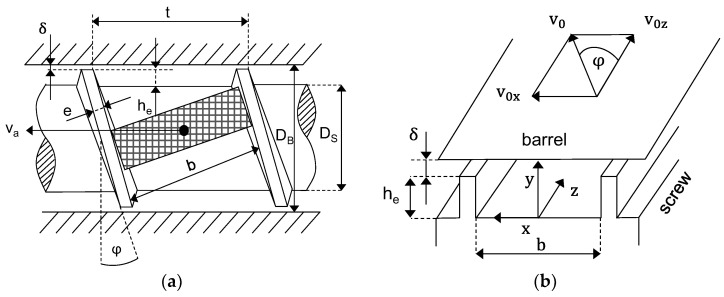
(**a**) Solid element in screw channel; (**b**) unwound channel with coordinate system fixed on screw.

In Figure 1a, the geometry of a screw is shown. The geometric parameters are the barrel diameter $D_{B}$, the screw core diameter $D_{S}$, the channel depth $h_{e}$, the screw pitch t, the screw clearance δ, the channel width b, the helix angle φ and the flight width e. The axial speed of the solid element in the conveying direction is indicated with $v_{a}$. This speed and its calculation differ depending on the approach and will be discussed later. In Figure 1b, the helical screw channel is unwound to a flat channel with a coordinate system fixed on the screw so that the barrel moves over the channel with a peripheral speed $v_{0}$ and its vector components $v_{0z}$ and $v_{0x}$ [1].

#### 1.1.1. Analytical Models

Due to the fact that analytical models for describing solids conveying in grooved barrel extruders are based on models for smooth barrel extruders, the latter are described first. This is followed by introducing special models for grooved barrel extruders.

**Figure 2:**
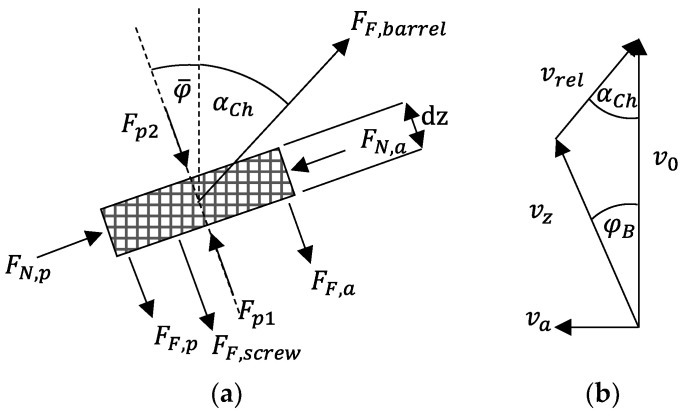
(**a**) Forces acting on solid bed; (**b**) balance and analysis of velocities in the screw channel.

Many of the approaches to describe solids conveying in a single-screw extruder that have become known up to now go back to the model concept of Darnell and Mol [2]. In this model, the assumption is made that the pellets behave as a block, in a similar manner to a rigid solid, and flow through the screw channel as a so-called solid bed in a block flow. As shown schematically in Figure 2a, various pressure and friction forces act on this solid bed, which allow the direction of movement to be calculated on the basis of a force and moment balance. Finally, the so-called channel conveying angle $\alpha_{Ch}$ is calculated, at which the material flows towards the tip of the screw. It is determined between the barrel peripheral speed $v_{0}$ and the relative velocity $v_{rel}$, as can be seen in Figure 2b. This conveying angle is then used to derive the velocity components of the solids bed in the channel $v_{z}$ and axial direction $v_{a}$.

The modeling, especially of the forces, has since been discussed and adapted in detail in many publications. In the following, some important publications are briefly discussed.

The approach of Darnell and Mol was improved by the work of Schneider, in which the pressure anisotropy present in bulk materials was considered, but a real screw pitch of greater than zero was neglected. The values for the so-called pressure anisotropy and friction coefficients were comprehensively determined experimentally by Schneider [3]. This model was extended by Ingen Housz to include screw pitches of greater than zero [4]. Furthermore, Schneider’s approach was taken up by Tadmor and Broyer and extended by an energy balance that takes into account the heat conduction into the solid bed [5,6]. The material parameter bulk density, previously assumed to be constant, was first modeled as pressure-dependent by Hegele and Langecker [7,8]. A phenomenological description of the friction coefficients, their influence on the solids transport and the bulk density of various polymer pellets was presented by Hwang and McKelvey, where the determined values were determined as a function of temperature and pressure under near-process conditions [9]. Furthermore, numerous publications by Hyun and Spalding have become known. In [10], among others, they presented a model that is based on that of Schneider, but which, in detail, applies the force $F_{N,a}$ differently. There, it acts on the solid bed at an additional angle. Furthermore, the energy balances from [5] are taken into account. Another solids conveying model was also presented by Potente and Jungemann, which includes the pressure and temperature dependence of the bulk density. Furthermore, in this model, the pressure anisotropy coefficients are no longer assumed to be constant, but—following an analytical derivation—are assumed to be a function of the geometry of the screw channel and the friction coefficients [11]. Since all models mentioned so far can calculate the temperature and pressure profile only one-dimensionally [12], a two-dimensional calculation of the solids conveying based on the finite difference method (FDM) was developed by Hennes. Friction coefficients were extensively determined experimentally as a function of temperature, pressure and velocity and then modeled. The pressure anisotropy was considered with the aid of a custom-defined friction parameter, which is the product of the friction coefficient and the pressure anisotropy coefficient. This coupling is justified because often only the product of these two quantities is needed for the calculation of solids conveying [13]. The approach developed by Hennes and the experimental findings were integrated into a three-dimensional solids conveying model by Imhoff. Furthermore, hints on the boundary conditions necessary to solve the equations were given. However, due to a lack of computer power, the model could not be implemented and validated [12].

The first applicable calculation models for grooved feed sections were presented by Rautenbach and Peiffer [14,15]. These are based on the theoretical description of solids conveying in smooth barrel extruders, as presented in [2,3], as well as on transfers of these theories made until then to grooved barrel extruders [16,17]. Accordingly, it is also assumed here that the plastic pellets completely fill the screw channel and are conveyed as a solid bed at a conveying angle $\alpha_{Ch}$.

A model-theoretical gap was closed by undertaking a clear differentiation between frictional and interlocking conveying mechanisms. This was possible by defining so-called conveying cases [18]. For this purpose, the dimensions of the pellets were compared with those of the grooves and the channel and a case differentiation was carried out, which resulted in the interlocking conveying cases 1a and 1b as well as the frictional conveying cases 2a and 2b. The conveying cases were supplemented by a calculation method for the bulk density present in the channel [19]. This was necessary because the dimensions of the screw channel are generally much smaller than those of the vessel for the standardized determination of the bulk density according to ISO 60 [20]. Consequently, an ideal packing density could not be achieved in the screw channel due to influences of the channel wall and the bulk density needed to be corrected to lower values. This correction was also necessary and justified because for the throughput determination in recognized calculation models, it is assumed that there is a region without a pressure gradient in the beginning of the feed section of grooved barrel extruders. Therefore, for the throughput determination, no compaction of the solids bed and, thus, no pressure dependence of the bulk density needed to be considered [18].

For the first time, Miethlinger presented equations with which a frictional calculation of the conveying angle is also possible for helical grooves. An effective surface ratio of the grooves to the barrel circumference is defined, which is obtained by integration along a path curve. Since this effective surface ratio and the conveying angle are interdependent, an iterative procedure is used to calculate the conveying angle [21]. The work of Kaczmarek again emphasized, on an experimental basis, the importance of the bulk density and the consideration of the geometric boundary conditions for the calculation of the solids conveying throughput [22]. The work of Michels focused on the optimization of the feed system of single-screw extruders with grooved feed sections. It was found that sufficiently long feed openings and an enlarged barrel inner diameter, a so-called feed pocket, ensure that the specific throughput of the solids conveying section can be kept constant over wide ranges of screw speed [23].

The model for describing the throughput of grooved barrel extruders by Potente was extended by Bornemann to include helical grooves. In contrast to Miethlinger, the distinction between interlocking and frictional conveying is retained, so that in case of interlocking conveying, the conveying angle is equal to the grooves’ angle [24].

#### 1.1.2. Numerical Investigation of Solids Conveying

The previously described development of analytical models for describing solids conveying based on mathematical–physical considerations came to a temporary end at the beginning of the 2000s, as numerical simulations based on the discrete element method (DEM) made their way into plastics technology. This was made possible primarily by improved computer performance, which enabled DEM simulations to be realized even on a scale relevant to plastics processing. The DEM, developed by Cundall and Strack [25], was originally developed for the simulation of molecular dynamics and has since found extensive use in process engineering, mechanical engineering, and geotechnical engineering [26].

A major advantage of DEM simulations is that fewer a priori assumptions have to be made about the flow behavior of the plastic pellets. The basis of DEM is to represent particles as spheres or particles composed of multiple spheres. To calculate interactions of these particles with other particles or geometries, they are not meshed, but a so-called virtual overlap δ is applied. Depending on this overlap and the boundary conditions, so-called contact models calculate contact forces in normal and tangential directions. These forces are then used to solve the momentum conservation equations and calculate new motion quantities. When these motion quantities are integrated over the simulation time step, new positions of the particles result, and thus, new virtual overlaps δ occur and the calculation cycle starts again [26]. The mathematics of the DEM are described in more detail in Section 2.3.

Essential work for the use of DEM in plastics engineering was published by the working group around Moysey and Thompson. First, the suitability of DEM for the simulation of solid conveying processes was generally established [26,27]. In these simulations, effects known from practice, such as the backflow of pellets into the hopper opening, can be observed. Likewise, DEM simulations can show that the block flow assumed in all analytical models is no longer present even at a peripheral speed of 0.25 m/s. In further publications, Moysey and Thompson investigated the influence of the material parameters of the coefficient of friction and coefficient of restitution, as well as the heat conduction in the pellets and the influence of the backpressure on the compaction of the solid bed. These further developments have been validated by experimental investigations and good agreements between simulation and experiment are shown throughout [28,29,30].

In [31], it was shown that DEM is basically suitable for describing the conveying behavior of plastic pellets in single-screw extruders even at high speeds. For this purpose, simulatively determined throughputs were compared with both analytical calculation methods and experimental values. The test stand consisted of a barrel made of PMMA and a shortened screw clamped to a lathe. The experimentally determined throughput at low speeds up to 200 rpm could be calculated very well using an analytical approach according to Schneider [3]. However, it was also shown that the flattening of the experimentally determined throughput curve could not be reproduced by the Schneider model. This is also to be expected, since the Schneider model does not include a speed-dependent degree of filling as a parameter. In contrast, the DEM simulations were able to reproduce the degressive behavior of the throughput curve very accurately [31].

Based on these findings, the DEM model was used to design an improved feed section geometry of a high-speed extruder with a screw diameter of 30 mm. A systematic analysis of the influence of the feed zone geometry on the solids conveying throughput was carried out with special consideration of high screw speeds up to a peripheral speed of 3 m/s. The geometric variations of the feed opening were the feed opening length, the width and the number of feed pockets. It was shown that the length of the feed opening has, by far, the greatest influence on throughput at high speeds. The previously widespread view that a feed opening length of 1.5 D is basically sufficient was disproved. In fact, the throughput at peripheral speeds of 3 m/s could be further increased by extending the opening up to 3 D [32]. A comprehensive modeling of solid throughput in smooth barrels that considers the pressure build-up was undertaken in [33]. Furthermore, effects known from practice, such as the circulation of pellets in the feed section, could be predicted with very good accuracy by means of DEM [34]. First DEM simulations on grooved feed sections were carried out by Bonten et al. Here, the conveying behavior could be reproduced well in principle. Existing deviations were attributed to the lack of a backpressure in the simulations [35]. Recently, the DEM was also used for analyzing the flow behavior of regrind as a recycling material in a conical and grooved feed section [36].

Due to the good suitability of the DEM for describing solids conveying in extruders, it is now used for evaluating existing analytical calculation approaches for grooved feed sections. The main weak point of the analytical models described in Section 2.1 is that they never deviate from the assumption of a block flow. Moreover, for this reason, the calculation of a conveying angle on this block might be imprecise if the assumption of block flow does not apply. Furthermore, it has to be checked whether the a priori distinction of conveying cases is useful or not. Finally, it is also examined whether the assumption of a pressure-independent throughput is appropriate.

## 2. Approach and Methods

The starting point was the construction of a central composite design (CCD) using the software Design Expert 12, Stat-Ease Inc. For this purpose, the test area was delimited, target variables were defined and the influencing factors were determined. For the analytical calculation, the Bornemann [24] model was used, which is applied in a practice-oriented manner in the software REX 16.0.1 (computer-aided extruder design, developed at KTP). For numerical simulation, the software EDEM 2021, Altair Engineering, was used. Based on numerical throughput simulations with EDEM, the assumptions of the backpressure-independent throughput, the classification of the solids conveying mechanisms into conveying cases and the formation of a block flow were checked. Finally, the analytical calculation accuracy was investigated by comparing the throughputs according to Bornemann and EDEM.

### 2.1. Design of Experiments

For the investigation of certain influencing factors, a fractional factorial CCD of experiments was used. The CCD plan had several advantages in this investigation. First, the variation of the influencing factors on five stages generated a broad data base. By setting the stage parameters appropriately, different conveying cases occurred. By varying the barrel diameter, machine sizes that are relevant to practice and industry were also directly investigated. This was considered more important than the exclusive study of machine sizes in laboratory environments. As a consequence, the minimum possible barrel diameter $D_{B}$ was 35 mm, since otherwise the combination of low dimensionless channel depth $h_{e}/D_{B}$ and pellet diameter $d_{p}$ would have led to test points that could not be simulated. Since the experimental effort increased drastically with an increasing number of influencing factors, not all possible influencing factors could be considered or varied. For this reason, they were limited to the most important influencing factors for which a high influence on the solids conveying process could be expected. The other factors remained constant (if necessary, related to a size, e.g., to the barrel diameter). Eight influencing factors were varied as shown in Table 1.

With *k* = 8 influencing factors and a reduction level of *p* = 2, the resolution level of the DoE was *V*. The central point needed to be repeated ten times for *k* ≥ 5 [37]. Although it would be expected that due to the simulation, there would be no scatter in the final result, it was always the same, and thus, repetition was not necessary. However, since the pellets were randomly placed in the hopper in EDEM, a new arrangement of the pellets resulted for each solid conveying process, causing the contact forces to vary and, thus, scattering the results [38]. Considering the repetitions recommended in the literature [37], the number of experimental points was calculated to be:

(1) $$N=2^{k-p}+2k+n_{0}=2^{8-2}+2\cdot 8+10=90$$

Different tribological behaviors of materials could be realized by selecting the step settings of the internal coefficient of friction. The barrel and screw friction coefficients were deliberately not included in the test plan as additional influencing factors, since otherwise, the number of test points would have increased drastically. However, by specifying a constant ratio or difference to the internal coefficient of friction, these were always indirectly varied as well, see end of section. It should be noted that in the evaluation, an effect could not necessarily be attributed to a specific coefficient of friction, but always only to the ratio or the respective combination of the coefficients of friction. The choice of the friction coefficient ratios was based on the material calibration tests in [39], with which subsequently good simulative and experimentally consistent throughput results could be achieved.

In addition to the varying influencing factors, the settings of particularly relevant constant influencing factors were noted:

1. Length of feed section $L=4\;D_{B}$;
2. Barrel coefficient of friction $\mu_{B}=\mu_{i}-0.2$;
3. Screw coefficient of friction $\mu_{S}=\mu_{B}/2.5$;
4. Groove shape: rectangular;
5. Groove width $b_{G}=8\;\mathrm{mm}$;
6. Groove depth $h_{G}=3\;\mathrm{mm}$, tapered towards the end of the feed section;
7. Number of grooves $\;N_{G}$ was calculated, so that one third of the barrel surface was covered with grooves [24]:

(2) $$\;N_{G}=\frac{1}{3}\cdot \frac{\pi \cdot D_{B}\cdot sin\left(\varphi_{G}\right)}{b_{G}}$$

### 2.2. Mathematics of Analytical Calculation

In the following, the most important equations of the analytical throughput model, according to Bornemann, are described. For all details, see [24]. Unless otherwise specified, the geometric quantities were evaluated at the front edge of the hopper, since this was also where the throughput was determined.

The total throughput ${\dot{m}}_{tot}$ of a grooved feed section is the sum of the throughput of the screw channel ${\dot{m}}_{Ch}$ and the grooves ${\dot{m}}_{G}$:

(3) $${\dot{m}}_{tot}={\dot{m}}_{Ch}+{\dot{m}}_{G}$$

The throughput in the channel and in the grooves can be determined using a corrected bulk density $\rho_{corr}$, the cross-sectional area A of the channel or grooves and the axial velocity of the solid bed $v_{a}$. For the throughput in the screw channel or in the grooves, respectively, the following applies:

(4) $${\dot{m}}_{Ch/G}=\rho_{b,corr,\;Ch/G}\cdot A_{Ch/G}\cdot v_{a,\;Ch/G}$$

The axial cross-sectional area of the channel $A_{Ch}$ forms an annular gap that is calculated as follows:

(5) $$A_{Ch}=\frac{\pi }{4}\cdot \left(D_{B}^{2}-D_{S}^{2}\right)-\frac{i\cdot e\;\cdot h_{e}}{\sin\left(\bar{\varphi }\right)}$$

The axial cross-sectional area of the grooves $A_{G}$ is derived from their depth, width and number:

(6) $$A_{G}=N_{G}\cdot b_{G}\cdot h_{G}$$

$v_{a,\;Ch}$ arises from the velocity $v_{z}$ in the down channel direction and the screw helix angle at the barrel wall $\varphi_{B}$:

(7) $$v_{a,\;Ch}=v_{z}\cdot \sin\left(\varphi_{B}\right)$$

The bulk density in the screw channel and in the grooves depends on both the material and the geometry of the feed section. The standard bulk density $\rho_{b,0}$ can be easily determined using the standardized ISO 60 method. However, the geometry used in this method is different from that in the feed section. The bulk density present decreases significantly due to influence effects of the channel wall. The pellet layers adjacent to the wall are less dense than those inside the bed of solids or the bulk material. This effect increases as the ratio of channel volume to pellet size decreases, and the corrected bulk density is correspondingly lower. For this reason, the corrected bulk density $\rho_{b,corr}$ is calculated with Equation (4). The values for depth h and width b are correspondingly inserted for the channel and groove.

(8) $$\rho_{b,corr}=\rho_{b,0}\cdot \frac{\left[\left(\frac{h}{d_{p}}\right)-1\right]\cdot \left[\left(\frac{b}{d_{p}}\right)-1\right]+\frac{1}{\sqrt{2}}\cdot \left[\left(\frac{h}{d_{p}}\right)+\left(\frac{b}{d_{p}}\right)-2\right]+\frac{1}{2}}{\left[\left(\frac{h}{d_{p}}\right)-1\right]\cdot \left[\left(\frac{b}{d_{p}}\right)-1\right]+\left(\frac{h}{d_{p}}\right)+\left(\frac{b}{d_{p}}\right)-1}$$

**Figure 3:**
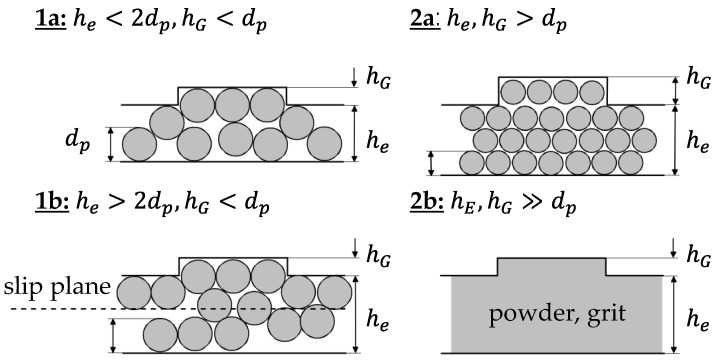
Illustration of the different conveying cases according to [18].

In the throughput Equation (3), only the velocities of the solids in the channel and in the grooves remain as unknown variables. Their calculation requires a more detailed consideration of the conveying mechanisms occurring in the process. Because of the direct connection of channel and groove, interactions can occur between them, which influence the respective velocities and, thus, a mutual dependence can exist. The conveying of the pellets is based on interlocking and frictional mechanisms. According to [18], the conveying mechanism depends on the pellet diameter $d_{p}$, the groove depth $h_{G}\;$and the channel depth $h_{e}$. In case 1a, pure interlocking conveying occurs, which is based on the form fit of the pellet in the groove and the screw channel. For this case, on the one hand, the diameter of the pellet must be larger than the groove depth and, on the other hand, there must be a shallow channel depth, with the condition $h_{e}<2d_{p}$. Both the pellets in the grooves and in the channel are then forced to be conveyed. Case 1b differs from case 1a in classification only in the ratio of the channel depth to the pellet diameter with the condition $h_{e}>2d_{p}$. This larger channel depth leads to the formation of a slip plane below the top layer in the screw channel. According to this model assumption, the pellets in the grooves and in the top layer in the channel are forced to be conveyed, while the remaining material in the channel below the slip plane is frictionally driven. In case 2a/b, the pellet or powder is smaller than the groove and channel depths, resulting in only frictionally driven conveying. The differentiation of the conveying cases is shown in Figure 3 [18].

This subdivision according to [18] is also used by Bornemann for his pressure-throughput modeling, whereby two states are defined for conveying case 1b. From a physical and theoretical point of view, the velocity of the pellet below the slip plane can only have two different states. One state is that the lower pellet layer adheres to the layer above it due to friction and, thus, moves at the same speed. The other state is that the friction in the slip plane is too low or too high at the base of the screw and, therefore, the material below the slip plane stagnates in the screw channel. According to Bornemann, the first condition is much more probable due to the high internal frictional forces compared to the low frictional forces at the screw base. With this justification, conveying case 1b is treated largely analogously to conveying case 1a [24].

Depending on the defined conveying cases, it is possible to calculate the speed at which the material moves in the screw channel or groove and how strongly the material is inhibited from rotating along with the screw.

Basically, the material flows along the screw channel at the velocity $v_{z}$ provided that the barrel transmits a force to the solid block at the peripheral speed $v_{0}$. Then, there is a relative velocity $v_{rel}$ between the barrel and solid block. $v_{rel}$ encloses the conveying angle $\alpha_{ch}$ with the peripheral direction. The better the force transmission, the larger it becomes. With a balance of all occurring velocities, see Figure 2b, the solid bed velocity can initially be calculated as follows:

(9) $$v_{z}=v_{0}\cdot \frac{\sin\left(\alpha_{Ch}\right)}{\sin\left(\alpha_{Ch}+\varphi_{B}\right)}$$

Finally, the channel conveying angle $\alpha_{Ch}$ is an unknown variable. For conveying cases 1a and 1b, the calculation is trivial, since the conveying angle is given by the groove angle, so that $\alpha_{Ch}=\varphi_{G}$. For conveying cases 2a and 2b, the conveying angle must be calculated based on a force and moment balance according to Figure 2a. The solid bed moving in the screw channel is in contact with the surfaces of the screw root, the screw flights as well as the barrel due to the internal pressure. This internal pressure is expressed by the normal forces. Due to the movement of the solid bed, frictional forces occur in the interfaces. For the normal forces on the screw flights, the assumption is made that the rotary motion of the screw causes the active flight to exert an additional force ΔF on the solid bed and that the passive flight relieves it by the same amount. Under the further assumption that the operating behavior is in a backpressure-independent range and, thus, there is no pressure gradient dp in the channel direction, the forces $F_{p1}$ and $F_{p2}$ are equal. In contrast, in the other forces the anisotropic pressure propagation in a bulk material must be taken into account by the pressure anisotropy coefficients, denoted by $k_{i}$. A further derivation of the conveying angle is not presented here; it can be found in Appendix B.

In conveying case 2a, the conveying speed in the channel and in the groove depends on the friction. Since the material in the channel and in the groove are in direct contact with each other, the occurring flows influence each other. Interactions therefore occur between the solid bed in the screw channel and the solid bed in the grooves, which must be included in the velocity balance. Depending on whether the groove angle is larger or smaller than the channel conveying angle, the solids bed velocity of the channel component increases or decreases. The groove flow changes the effect of the barrel peripheral velocity. The original channel conveying angle remains unaffected by this interaction. Depending on the solids bed velocity in the grooves, a new solids bed velocity in the channel $v_{z,\;new}$ results:

(10) $$\begin{array}{c} v_{z,new}=v_{0}\cdot \frac{\sin\left(\alpha_{Ch}\right)}{\sin\left(\alpha_{Ch}+\varphi_{B}\right)}\\ +{\bar{v}}_{G}\cdot \left[\frac{\sin\left(\varphi_{G}\right)}{\sin\left(\varphi_{B}\right)}-\frac{\sin\left(\alpha_{Ch}\right)}{\sin\left(\alpha_{Ch}+\varphi_{B}\right)}\cdot \left(\cos\left(\varphi_{G}\right)+\frac{\sin\left(\varphi_{G}\right)}{\tan\left(\varphi_{B}\right)}\right)\right] \end{array}$$

Since the groove flow influences the channel flow directly below the grooves, the groove velocity must be averaged over the barrel circumference. For this reason, the average groove velocity ${\bar{v}}_{G}$ is used in Equation (10). It is calculated as follows:

(11) $${\bar{v}}_{G}=v_{G}\cdot \frac{N_{G}\cdot b_{G}}{\pi \cdot D_{B}\cdot \sin\left(\varphi_{G}\right)}$$

The grooves velocity $v_{G}$ is derived with a velocity balance in an analogous approach as for the channel velocity. If the groove angle matches the channel conveying angle ($\varphi_{G}=\alpha_{Ch}$), the following applies to the groove velocity:

(12) $$v_{G}=v_{0}\cdot \frac{\sin\left(\varphi_{B}\right)}{\sin\left(\varphi_{B}+\varphi_{G}\right)}$$

Provided that the channel conveying angle does not match the groove angle ($\varphi_{G}\neq \alpha_{Ch}$), the following applies:

(13) $$v_{G}=\frac{\sin\left(\varphi_{B}\right)\cdot \left[v_{0}\cdot \sin\left(\alpha_{G}\right)-v_{Ch}\cdot \sin\left(\varphi_{G}+\alpha_{G}\right)\right]}{a+b-c}$$

with

(14) $$a=\sin\left(\alpha_{G}\right)\cdot \sin\left(\varphi_{B}\right)\cdot \cos\left(\varphi_{G}\right)$$

(15) $$b=\sin\left(\alpha_{G}\right)\cdot \sin\left(\varphi_{G}\right)\cdot \cos\left(\varphi_{B}\right)$$

(16) $$c=\sin\left(\varphi_{G}\right)\cdot \sin\left(\varphi_{B}+\alpha_{G}\right)$$

Due to the interactions between the channel and the groove flow in conveying case 2a, the velocities must be calculated iteratively until they converge. For this purpose, the solid bed velocity $v_{z}$ in the channel without interactions is determined as the initial velocity. This can then be used to calculate the first groove velocity $v_{G}$. The averaged groove velocity is used to calculate the new channel velocity $v_{z,new}$. This new channel velocity is compared with the original one. If these do not converge, the calculation step starts again with $v_{z,new}$ as the next iteration start value.

At last, the grooves conveying angle $\alpha_{G}$ has to be calculated. Similar to the channel conveying angle, a force balance is established here, which leads to the following determining equations:

(17) $$\alpha_{G}=\varphi_{G}-arccos\left(\frac{\mu_{B}}{{\bar{\mu }}_{i}}\cdot \left(1+2k\cdot \frac{h_{G}}{b_{G}}\right)\right)for\;\varphi_{G}>\alpha_{Ch}$$

(18) $$\alpha_{G}=\varphi_{G}+arccos\left(\frac{\mu_{B}}{{\bar{\mu }}_{i}}\cdot \left(1+2k\cdot \frac{h_{G}}{b_{G}}\right)\right)for\;\varphi_{G}<\alpha_{Ch}$$

As in the screw channel, an average internal coefficient of friction ${\bar{\mu }}_{i}$ must be used in the interface below the groove. Since the screw flight partially runs below the grooves, not only the internal coefficient of friction acts, but also that of the screw. The following thus applies to the average internal coefficient of friction ${\bar{\mu }}_{i}$:

(19) $${\bar{\mu }}_{i}=\frac{b\cdot \mu_{i}+e\cdot \mu_{S}}{b+e}$$

With the equations described up to this point, the throughput can be determined. The calculation of the throughput in conveying case 2b can be carried out analogously to conveying case 2a. An empirical equation for the mean barrel friction coefficient can be considered, which includes a cross flow in the grooves [40]. However, this conveying case is not examined in this work.

### 2.3. Numerical DEM Simulation Model

The discrete element method belongs to the numerical calculation methods and is suitable for simulating the movement of the pellets in a process under certain assumptions. This includes that the polymer pellets are considered as rigid spheres in a simplified way. Although it is possible to approximate real pellet geometries by assembling individual pellets, this drastically increases the simulation time. This is due to the calculation method of the DEM. The starting points of the calculation are the contact events between the stationary or moving spheres with their environment (other spheres or the geometry). From these contacts, the acceleration, velocity and position of the spheres can be determined. Taking into account the interactions in the contact surfaces and the material properties, this is achieved by setting up and solving the momentum balance. In the DEM, both the translational and rotational movements of the spheres are determined [41,42]:

(20) $$m_{i}\frac{d{\vec{v}}_{i}}{dt}=\underset{j}{\sum }\left({\vec{F}}_{n,\;ij}+{\vec{F}}_{t,ij}\right)+m_{i}\cdot \vec{g}$$

(21) $$I_{i}\frac{d{\vec{\omega }}_{i}}{dt}=\underset{j}{\sum }{\vec{M}}_{ij}$$

**Figure 4:**
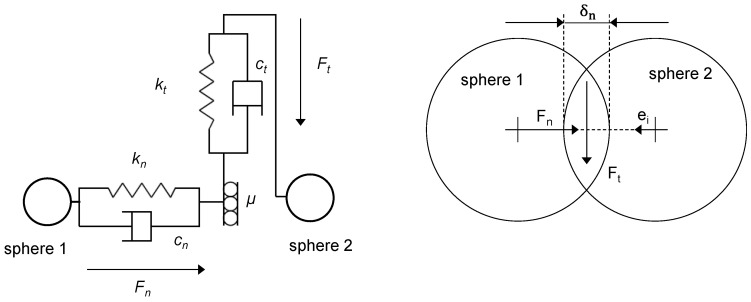
Representation of the spring-damper system (**left**) based on the contact of two spheres (**right**) in the DEM.

The forces occurring in the contact thus form the basis of all further movement of the spheres. The way in which these forces are calculated depends on the contact model selected. The basis of all contact models is that the spheres are assumed to be rigid. Nevertheless, in order to be able to consider real deformations, these are represented as virtual overlapping of the spheres in the normal and tangential directions, $\delta_{n}$ and $\delta_{t}$, respectively. The overlap results from the integration of the velocity over the time step and the new positions of the spheres. A frequently used and proven contact model is the Hertz–Mindlin contact model. In this model, elastic and damping force components are included for the deformations. For this purpose, this contact model uses the physical model of the spring-damper system. The total tangential force is limited to the friction coefficient μ. Figure 4 schematically illustrates the contact of two spheres and the spring-damper system used in the contact model. The calculation of the spring and damping constants k and c, respectively, is contact-model-specific and is based on the material input parameters. For the Hertz–Mindlin contact model, it is well documented and can be found, for example, in [38,41,42].

The process of calculating the contact forces or the motion of the spheres takes place iteratively in discrete time steps. At each time step, all contact forces are first calculated for each sphere and then all Newtonian equations of motion are solved. The whole process is repeated until a certain termination criterion is reached, usually when the total simulation time is reached. Depending on the material properties, different discrete time steps are required for the calculation. These have a decisive influence on the calculation accuracy of the movement of the spheres. The smaller they are selected, the more accurately the forces can be calculated. However, smaller time steps also require more calculation steps and, thus, the simulation duration increases equally. On the other hand, time steps that are selected too large lead to unrealistic overlaps, which then result in contact forces or movements that are too large. The mathematical criterion for calculating the optimum time step is based on the propagation velocity of interfering waves, so-called Rayleigh waves. These waves cause the problem that the energy transfer is not only limited to the directly neighboring spheres, but also to spheres further away. For this reason, the critical time step for a stable simulation is determined based on the propagation velocity of Rayleigh waves. This critical time step$\;t_{Rayleigh}$ results from the minimum sphere radius $R_{min}$, the density $\rho_{min}$, the shear modulus $G_{max}$ and the Poisson ratio $\nu_{max}$ [41]:

(22) $$t_{Rayleigh}=\frac{\pi \cdot R_{min}\cdot \sqrt{\frac{\rho_{min}}{G_{max}}}}{0.1631\cdot \nu_{max}+0.8766}$$

To apply a backpressure in the simulations, a force field at the end of the feed section is applied. Being connected with the EDEM API, it is implemented as a custom particle body force model. Every particle that is located in that defined field is loaded with an additional constant force $F_{p}$ that acts parallel to the screw axis and against the conveying direction. It is calculated from the geometrical parameters of the screw and the number of particles $n_{p}$ within the force field according to Equation (23). This procedure was developed and validated in [42].

(23) $$F_{p}=\frac{p\cdot A}{n_{p}}=\frac{p\cdot \pi \cdot \left(D_{B}^{2}-D_{S}^{2}\right)}{4\cdot n_{P}}$$

**Figure 5:**
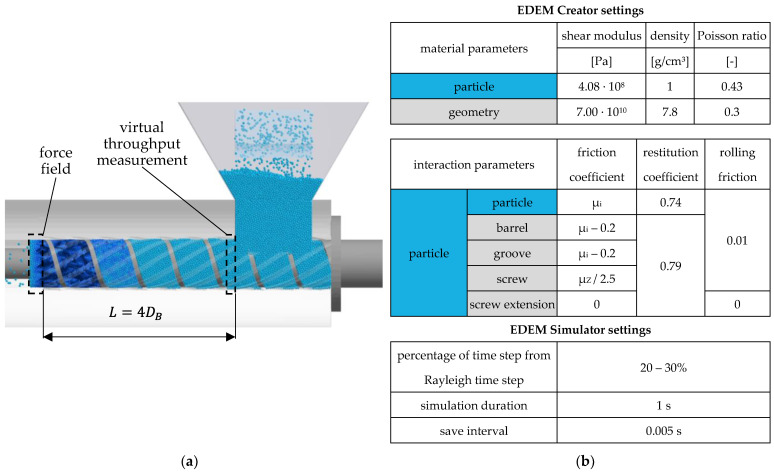
**(a)** Representation of a simulation model in EDEM; (**b**) summary of the main simulation settings in EDEM.

In Figure 5a, the simulation model is shown for an exemplary test point. For good comparability with the analytical calculation, the throughput, axial velocity and bulk density at the front edge of the hopper are each evaluated separately for the screw channel and the grooves. Furthermore, the entire feed section is divided into 20 so-called geometry bins in order to evaluate the pressure on the screw and barrel as a function of the length. Figure 5b summarizes the most important simulation settings used in EDEM. These are based on findings from experimental throughput and compression tests, where good agreement between simulations and experiments could be achieved [39].

As shown in Section 2.2, the conveying angle $\alpha_{Ch}$ is a well-suited quantity to describe the effectiveness of solids conveying in an abstracted way. Since it is not available as a direct variable in the numerical simulations, a numerical channel conveying angle $\alpha_{Ch,num}$ must be calculated from the mean axial velocity in the channel ${\bar{v}}_{a,\;Ch}$:

(24) $$\alpha_{Ch,num}=arctan\left(\frac{{\bar{v}}_{a,\;Ch}\cdot tan\left(\varphi_{B}\right)}{v_{0}tan\left(\varphi_{B}\right)-{\bar{v}}_{a,\;Ch}}\right)$$

## 3. Results, Analysis and Discussion

In the following sections, the test points from analytics and EDEM are examined, compared with each other and the resulting differences are analyzed.

The first step is to check to what extent the assumptions made for the analytical calculation can be observed in EDEM. These include the assumptions of a backpressure-independent throughput, the subdivision of the test points into the conveying cases 1a, 1b and 2a, and the assumption of a block flow in which there is no relative movement of the pellets. Based on these findings, improvement of the analytical modeling is suggested.

In the second step, the improved analytical calculation accuracy is checked in comparison with the numerical simulation. The simulated solids conveying using EDEM represents real throughput tests on a grooved barrel extruder under the limitations described in Section 2.3.

### 3.1. Investigation of Assumptions: Backpressure Independence

An essential assumption in the analytical calculation of the throughput is that there is a region at the beginning of the feed section in which there is no pressure gradient in the direction of the screw channel. Only then is the throughput independent of backpressure and, thus, can be determined using the analytical equations described in Section 2.2 [24].

**Figure 6:**
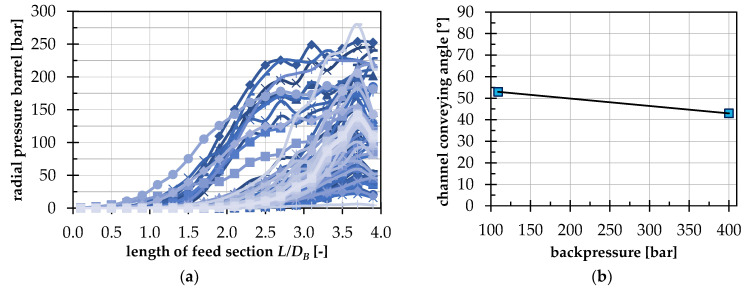
(**a**) Pressure curve on the barrel for all 90 test points; (**b**) influence of the backpressure on the conveying angle as an effect diagram from the analysis of variance with Design Expert.

On the basis of Figure 6a, the pressure at the barrel can be observed for all 90 test points. It should be noted that the measured pressure at the barrel does not correspond to the pressure in the channel direction, since the pressure in a bulk material is distributed anisotropically [3]. The pressure at the barrel only reflects the tendency of the pressure growth in the direction of the screw tip. For this reason, it is also possible that the radial pressure at the barrel at 4 $L/D_{B}$ is smaller than the set backpressure at the end of the feed section. In general, it should be noted that the pressure does not increase sharply in the area of the front edge of the hopper in the range of 0 to 0.2 L/D. The larger array of curves shows an exponential increase in pressure in the range of 1.7 to 2.7 L/D with a maximum at about 3.7 L/D. The exponential pressure curve fits the accepted calculation models [24]. To investigate the effect of backpressure on the conveying behavior in more detail, the conveying angle was calculated from the EDEM simulations and subjected to an analysis of variance using Design Expert. Figure 6b shows how the conveying angle changes as a function of the backpressure steps from the selected experimental design. The mean change from $\alpha_{Ch}=53{}^{\circ}$ at 100 bar backpressure to $\alpha_{Ch}=43{}^{\circ}$ at 400 bar backpressure can be classified as moderate. This applies especially against the background that modern grooved barrel extruders are usually designed with a neutral pressure profile, so that the pressure in the grooved barrel is reduced [43].

### 3.2. Investigation of Assumptions: Conveying Cases

**Figure 7:**
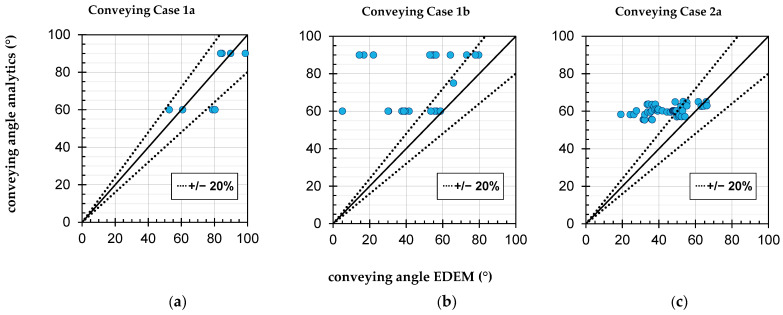
Conveying angle from the analytical calculation according to Bornemann plotted against the conveying angle from the EDEM simulations, sorted by conveying cases 1a, 1b and 2a.

According to the model presented in Section 2.2, different conveying cases can occur during conveying in the grooved barrel extruder. The aim was to check whether the assumption of these conveying cases is justified from the point of view of the EDEM simulations. In Figure 7, the conveying angle according to the analytical calculation is compared with that from the DEM simulations. For case 1a, see Figure 7a, it can first be seen that the conveying angles, according to the Bornemann model, correspond to the selected steps of the groove angles of 60° and 90°. The EDEM values do not deviate much from this. The slight overestimations in the EDEM conveying angles are mainly due to the fact that slight partial fills and space changes occur sometimes. These provide a short-term acceleration of the particles and shift the mean axial velocity, and thus, the conveying angle according to Equation (24), to higher values. The assumption of case 1a is, therefore, justified for the analytical calculation.

The situation in case 1b, see Figure 7b is much less clear. Of the 25 test points, 8 show a small deviation of well below 20%, 13 deviate slightly more than 20% and 4 deviate strongly. A visual analysis of the latter test points, which are described in detail in [44], shows that there is no interlocking forced conveying, neither in the channel nor in the grooves. Obviously, the protrusion of the particles from the grooves is not sufficient for the screw flight to drive the particles. The protrusion results—according to the particle diameter of 3.39 mm, a groove depth of 3 mm and a screw clearance of 0.2 mm—in a value of 0.19 mm. At this point, it is interesting to compare the results with those of case 1a, where no such phenomenon can be observed despite the same geometrical boundary conditions between grooves and particles. Due to the small number of test points deviating strongly, it cannot be clearly determined which of the influencing factors from the test plan is causal for the slippage. However, sample investigations of test point 64 (see Appendix C for details) show that even increasing the particle diameter from 3.39 mm to 4 mm greatly improves the form fit [44]. By these means, the numerical conveying angle rises from 14° to 48° and the total numerical throughput rises from 461 kg/h to 828 kg/h. The analytical throughput only changes insignificantly from 1154 kg/h to 1128 kg/h due to a slight reduction in the corrected bulk density. In view of the fact that real pellets, due to their shape deviating from an ideal sphere, always also have a form-fit component of the force transmission, the assumption of case 1b according to Bornemann also appears justified as an approximation. Nevertheless, the formation of a slip plane is possible from a numerical point of view; however, a prediction of it by means of an analytical criterion seems difficult.

For case 2a, see Figure 7c, it can basically be stated that the assumption of frictional conveying is justified. However, it can also be seen that the analytical model calculates only a slight variation of the conveying angle in the range of approx. 55° to 65°, whereas the numerically determined conveying angles scatter considerably more with the same factor settings of the DoE. Since 57 test points are available for case 2a, an analysis of variance with Design Expert is feasible and is described in Section 3.4.

### 3.3. Investigation of Assumptions: Block Flow

**Figure 8:**
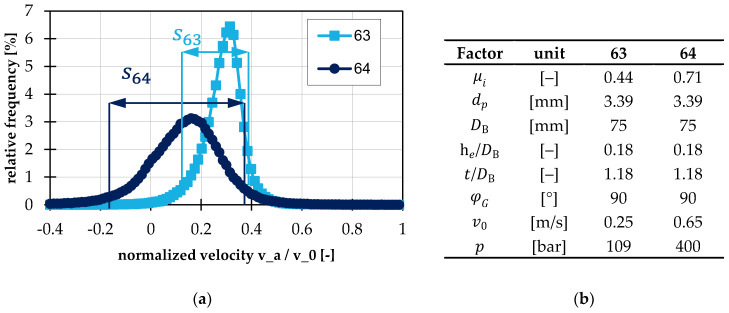
(**a**) Frequency distribution of normalized axial velocity with schematic illustration of decile distance s; (**b**) factor settings of test points 63 and 64.

During the visual analysis of the simulations, it became clear that the flow behavior differs significantly depending on the test points and their corresponding factor settings. For further evaluation, a mathematical criterion was developed that describes how much the flow is similar to or deviates from a block flow. For this purpose, the axial velocity of all particles in the first geometry bin ($L/D_{B}=0.2)$ was normalized to the peripheral velocity and its frequency distribution was determined. The distance between the first and the ninth decile was used as a measure of the spread of these velocities because it is insensitive to outliers, in contrast with the standard deviation, for example. This measure will be referred to as decile distance s in the following. The greater this decile distance, the more the flow deviates from a block flow. The evaluation and interpretation of this decile distance is shown as an example in Figure 8a. Here, the frequency distributions of the test points 63 and 64 are shown. These differ only in the factor settings of the internal coefficient of friction, peripheral velocity and backpressure, see Figure 8b. As can be seen, the spread differs significantly. It is approx. 0.25 for test point 63 and approx. 0.48 for test point 64.

**Figure 9:**
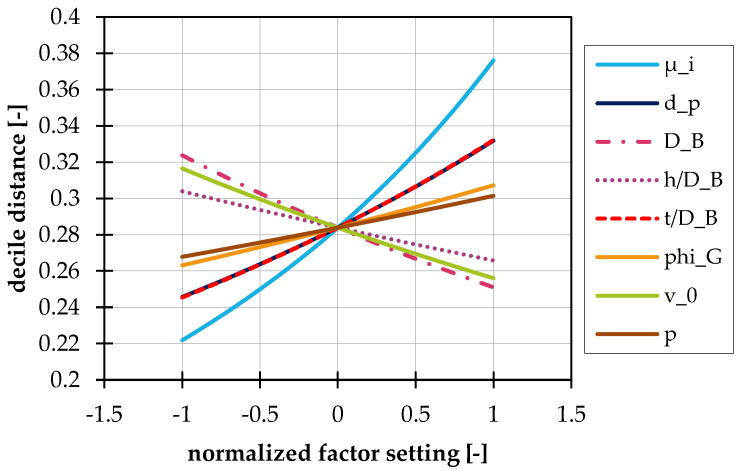
Perturbation plot of the decile distance of the normalized axial velocity in dependence on the normalized factor setting.

For further analysis, the decile distance of all test points was subjected to an analysis of variance. The result is shown in Figure 9. It can be seen that the inner coefficient of friction has the biggest influence on the decile distance s. In other words, the larger the internal coefficient of friction, the greater the deviation from the block flow. The same tendency can be seen for the pitch and the particle diameter. Further investigations for correlations between the decile distance and deviations in the conveying angle calculation showed no clear influence. There are test points where the deviation from the block flow is large and the calculation of the conveying angle is good, as well as the reverse case. From this point of view, a regression-based correction of the conveying angle seems to be of utility in terms of improving the throughput calculation. This is detailed in the following Section.

### 3.4. Modeling of a Correction Factor for Conveying Angle in Case 2a

Based on the findings in Section 3.3, a straightforward regression-based correction factor was developed for conveying case 2a. The variance analysis was not carried out with the numerical conveying angle itself, but on a correction factor. This has the advantage that the basically justifiable physical influences of the analytical description model were retained and were merely corrected with a regressed factor $f_{C,\;Reg}$. This factor is defined as follows:

(25) $$f_{C,\;Reg}=\frac{\alpha_{Ch,num}}{\alpha_{Ch}}=function\left(\frac{h_{e}}{D_{B}},\frac{t}{D_{B}},\;\varphi_{G},\;v_{0}\;\right)$$

The following regression equation is obtained:

(26) $$\begin{array}{c} f_{C,\;Reg}=\\ 0.884838-\left(1.80209\cdot \frac{h_{e}}{D_{B}}\right)-\left(0.269007\cdot \frac{t}{D_{B}}\right)+\left(0.003557\cdot \varphi_{G}\frac{1}{{}^{\circ}}\right)\\ +\left(0.093772\cdot v_{0}\cdot \frac{s}{m}\right) \end{array}$$

**Figure 10:**
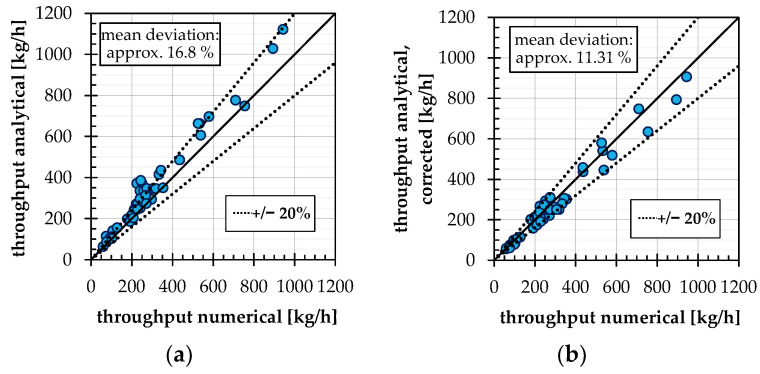
(**a**) Comparison of the numerically determined throughputs in conveying case 2a with the analytical model according to Bornemann; (**b**) comparison of the numerically determined throughputs in conveying case 2a with the analytical model according to Bornemann, corrected with regression factor $f_{C,\;Reg}$.

If this correction factor is used in the analytical calculation of the conveying angle according to Bornemann, the average deviation of the total mass flow rates for case 2a calculated in this way is reduced from 16.8% to 11.31%. This is shown in Figure 10.

### 3.5. Adjustment of Classification of Conveying Cases

**Figure 11:**
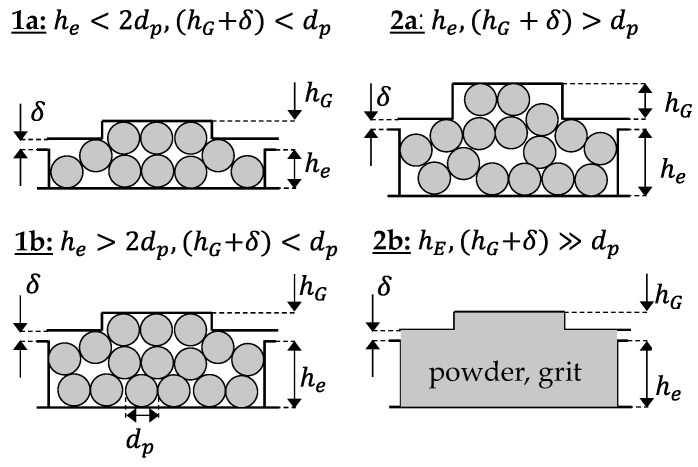
Adjustment of the definition of the conveying cases by the screw clearance δ.

For some test points of conveying case 1b, it was observed that the pellet in the groove slides over the screw flight, although according to Bornemann the pellet diameter is larger than the groove depth and thus interlocking conveying should occur. Assuming that the pellet does not deform or shear off, the previous definition is insufficient from a theoretical point of view. The decisive factor for forced conveying is whether the pellet protrudes from the groove and the screw flight drives it positively. If, however, the screw clearance is greater than the protrusion of the pellets from the groove, the pellets will merely slide over the screw flight. Especially if the combination of pellet diameter and groove depth is on the limit between conveying cases 1 or 2, the screw clearance can have a decisive influence on the model. The definition of the conveying cases according to Bornemann is extended by the consideration of the screw clearance, shown in Figure 11. Here, the criterion for forced conveying in the grooves is $\left(h_{G}+\delta \right)<d_{p}$ instead of $h_{G}<d_{p}$. For the test points defined here in the test plan; however, the assignment to the conveying cases does not change.

## 4. Summarizing Comparison of the Throughputs and Validation

**Figure 12:**
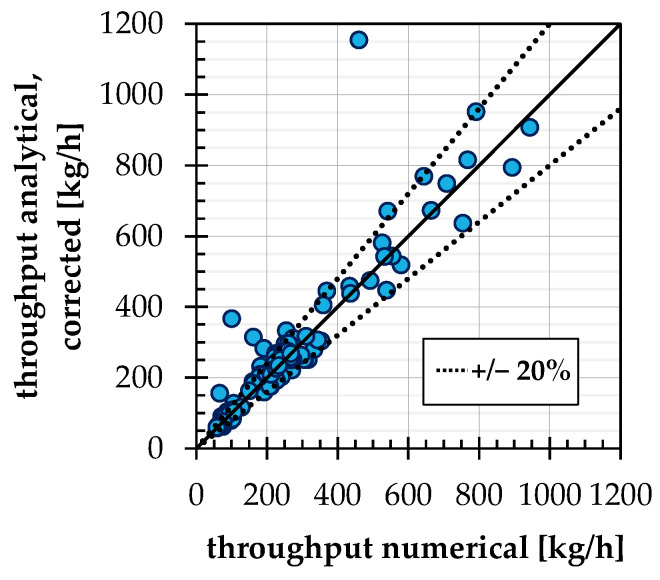
Comparison of numerical throughput with analytical calculation (with the use of the correction factor for case 2a) for all test points of DoE.

Finally, for all test points and conveying cases, the numerically determined throughputs are now compared with the analytical calculation with the changes mentioned above. The result is shown in Figure 12. As expected, the partly large deviations in the conveying angle of conveying case 1b, see Section 3.2, affect the throughput calculation. The average deviation across all test points is approx. 18.9%. If the four test points of conveying case 1b with particularly large deviations discussed in Section 3.2 are removed, the average deviation is reduced to 12.3%.

It can be summarized that the analytical calculation model modified in this way is basically very well suited to quickly calculate throughputs of grooved barrel extruders. However, it can also be seen that analytical equations reach their limits when complex flow phenomena occur, such as slippage of individual layers or stagnation. To further analyze such phenomena, a direct investigation with DEM seems suitable in the future. Since even very small changes in the particle and resulting bulk material properties determine whether slippage occurs, a correspondingly very accurate calibration of the bulk material behavior is essential.

**Figure 13:**
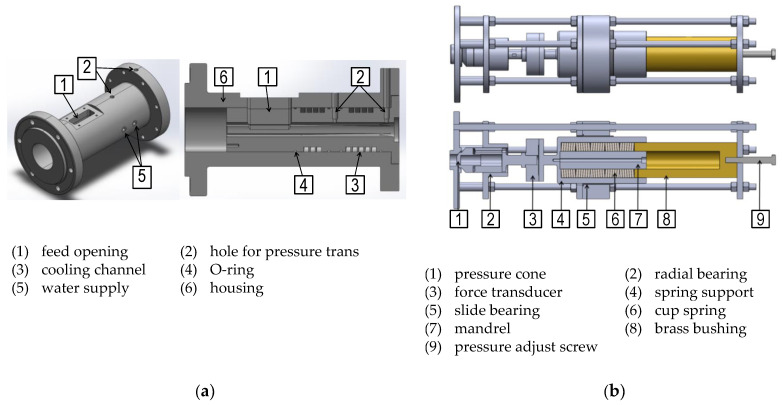
(**a**) Isometric and sectional view of the shortened filler housing including grooved barrel; (**b**) side and sectional view of the backpressure device.

To further validate the calculation accuracy of the analytical throughput model for practical applications, experimental investigations were carried out on a special solids conveying test bench. This consists of a shortened single-screw extruder with a grooved feed section and a backpressure device with which defined backpressures can be applied at the end of the solids conveying section. It has a nominal diameter $D_{B}$ of 30 mm, a length of 6 $L/D_{B}$ and four rectangular axial grooves ($\varphi_{G}=90\;{}^{\circ}$) with a width of 8 mm and a depth of 3 mm, tapered to the end of the feed section. The schematic structure of the setup is shown in Figure 13. The backpressure device basically consists of a cone that closes the outlet of the feed section. This cone is preloaded with cup springs and an adjustment screw. The resulting force is measured with a transducer. Various combinations of series and parallel connections of the cup springs allow the stiffness of the entire spring package to be flexibly adjusted.

The experiments were carried out with six different materials, three different screws, three different backpressures and three screw speeds resulting in a sum of 162 test points. The materials are a linear low-density polyethylene LL6301 (LLDPE), ExxonMobil, a polyamide 6 B 40 FA (PA6), Lanxess, a polypropylene RD204CF (PP), Borealis and a polystyrene 124 N (PS), Styrolution, in three different pellet sizes. The corresponding average pellet sizes $d_{p}$ are 0.99 mm (hereafter referred to as small), 1.36 mm (medium) and 2.86 mm (large). The other varied parameters, in detail, as follows:

- Screw speed: 70 rpm, 285 rpm and 500 rpm;
- Backpressure: 0 bar, 110 bar and 220 bar (LLDPE, PA6, PP);
- Backpressure: 0 bar, 7 bar and 14 bar (PS);
- Screw 1: t=1 D; $h_{e}=5.5\;\mathrm{mm}$; e=3 mm;
- Screw 2: t=1.2 D; $h_{e}=5.5\;\mathrm{mm}$; e=3 mm;
- Screw 3: t=1 D; $h_{e}=7.5\;\mathrm{mm}$; e=3 mm.

For all test points, the throughput was measured by means of a collecting vessel and a balance. The corresponding analytical throughput calculations were conducted using the model described in Section 2.2, whereby an automatic differentiation into conveying cases according to the definition in Section 3.5 is made. All necessary material data can be found in the Appendix D.

**Figure 14:**
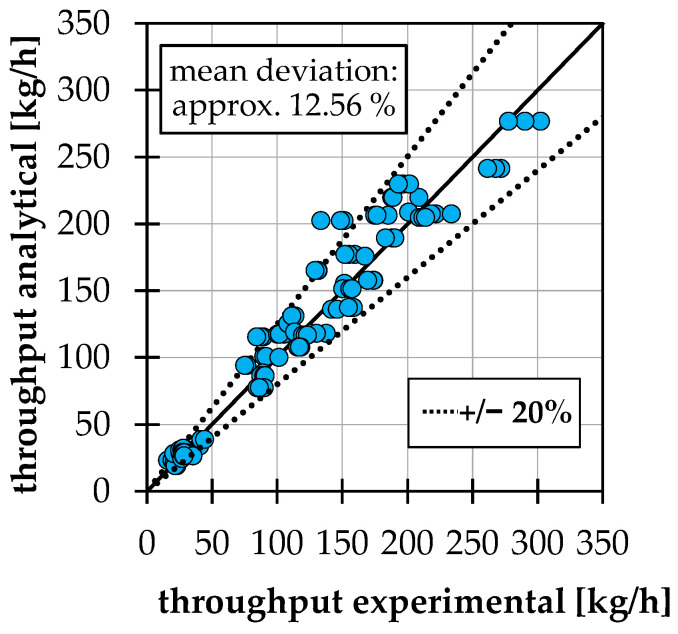
Comparison of experimental throughput with analytical calculation (without use of correction factor for conveying case 2a).

In Figure 14, the experimentally determined throughputs are compared to the analytical calculation. Since the barrel diameter of the extruder is 30 mm, which is out of the range of the DoE in Section 2.1, the correction factor $f_{C,\;Reg}$ is not used. The results show that there is good agreement between the experiments and the analytical calculation.

## 5. Conclusions and Outlook

Based on a design of experiments, the accuracy of the analytical throughput model of Bornemann was checked with numerical simulations using the discrete element method. Particular focus was placed on the assumptions made in the analytical throughput calculation, which are a pressure-independent throughput, the assumption of a block flow and the division of the solids conveying into the conveying cases 1a, 1b and 2a. It was shown that the assumption of a pressure-independent throughput and the division into conveying cases is justified. The assumption of a block flow is often not valid, even though this does not inevitably lead to large deviations in the calculated conveying angle. Based on these findings, a regression-based correction factor for conveying case 2a was developed. The validation tests confirm the basically good suitability of the analytical throughput calculation.

## Figures and Tables

**Table 1:** Factors and step values of central composite design.

Factor	Meaning	Unit	−α	−1	0	+1	+α
$\mu_{i}$	inner coefficient of friction	-	0.35	0.44	0.58	0.71	0.8
$d_{p}$	particle diameter	mm	1	1.61	2.5	3.39	4
$D_{B}$	barrel diameter	mm	35	45	60	75	85
$h_{e}/D_{B}$	channel depth	-	0.1	0.12	0.15	0.18	0.2
$t/D_{B}$	screw pitch	-	0.7	0.82	1	1.18	1.3
$\varphi_{G}$	groove angle	°	50	60	75	90	100
$v_{0}$	peripheral speed	m/s	0.11	0.25	0.45	0.65	0.79
p	backpressure	Bar	10	110	255	400	500

## Data Availability

Not applicable.

## Appendix A. Nomenclature

**Table A1:** Roman Characters.

Character	Meaning
$A_{Ch}$	axial cross-sectional area of channel
$A_{G}$	axial cross-sectional area of grooves
$b_{G}$	width of grooves
b	channel width
$c_{N}$	damping constant in normal direction
$c_{T}$	damping constant in tangential direction
C	geometric variable for conveying angle calculation
$D_{B}$	barrel diameter
$D_{S}$	screw core diameter
$d_{p}$	particle diameter
$e_{i}$	unit vector of contact area of colliding particles
E	geometric variable for conveying angle calculation
e	flight width
${\vec{F}}_{n,\;ij}$	normal force vector of DEM collision
${\vec{F}}_{t,ij}$	tangential force vector of DEM collision
$F_{F,\;barrel}$	resulting frictional force from barrel
$F_{F,\;screw}$	frictional force from screw
$F_{F,a}$	frictional force from active flight
$F_{F,p}$	frictional force from passive flight
$F_{N,a}$	normal force from active flight
$F_{N,p}$	normal force from passive flight
$F_{N}$	normal force on particles
$F_{T}$	tangential force on particles
$F_{p}$	additional force for backpressure
$F_{p1}$	normal force from pressure
$F_{p2}$	normal force from pressure
$f_{C,\;Reg}$	regression-based correction factor
$G_{max}$	maximum shear modulus of DEM particles
$\vec{g}$	gravity vector
$h_{G}$	depth of grooves
$h_{e}$	channel depth
$I_{i}$	moment of inertia
i	number of flights
$k_{N}$	spring constant in normal direction
$k_{T}$	spring constant in tangential direction
K	geometric variable for conveying angle calculation
*k*	number of factors in DoE
$k_{1}$	pressure anisotropy coefficient on barrel
$k_{2}$	pressure anisotropy coefficient on screw flights
$k_{3}$	pressure anisotropy coefficient on screw root
${\vec{M}}_{ij}$	torque on DEM particle
${\dot{m}}_{Ch}$	channel mass throughput
${\dot{m}}_{G}$	groove mass throughput
${\dot{m}}_{tot}$	total mass throughput
m	mass
$\;N_{G}$	number of grooves
$n_{p}$	number of particles (in force field)
N	number of test points in DoE
p	backpressure; reduction level of DoE
$R_{min}$	minimum radius of DEM particles
t	screw pitch; time
$t_{Rayleigh}$	Rayleigh time step
${\bar{v}}_{G}$	average velocity of solids in grooves
${\vec{v}}_{i}$	velocity vector of DEM particle
$v_{0}$	peripheral speed
$v_{0x}$	peripheral speed in x-direction
$v_{0z}$	peripheral speed in z-direction
$v_{a,\;Ch}$	axial velocity of solids in channel
$v_{a}$	axial velocity
$\nu_{max}$	maximum Poisson ratio of DEM particles
$v_{rel}$	relative velocity
$v_{z,new}$	solid bed velocity in z-direction after iteration
$v_{z}$	solid bed velocity in z-direction

**Table A2:** Greek Characters.

Character	Meaning
$\alpha_{Ch,num}$	conveying angle obtained from numerical simulation
$\alpha_{Ch}$	channel conveying angle
$\alpha_{G}$	groove conveying angle
δ	screw clearance; virtual overlap
ΔF	unknown additional force
${\bar{\mu }}_{B}$	average barrel friction coefficient
$\mu_{B}$	barrel coefficient of friction
${\bar{\mu }}_{i}$	average internal coefficient of friction
$\mu_{i}$	internal coefficient of friction
$\mu_{S}$	screw coefficient of friction
$\nu_{max}$	maximum Poisson’s ratio of DEM particles
$\rho_{b,0}$	standard bulk density
$\rho_{b,corr}$	corrected bulk density
$\rho_{min}$	minimum density of DEM particles
φ	helix angle in general
$\varphi_{B}$	helix angle at barrel
$\varphi_{G}$	groove angle
$\bar{\varphi }$	mean helix angle
$\varphi_{S}$	helix angle at screw
${\vec{\omega }}_{i}$	angular velocity of DEM particle

## Appendix B. Detailed Mathematics for Calculation of Conveying Angle

As a simplification, it is assumed that due to the small channel depth compared to the large pellets, the pressure is constant over the channel depth and, thus, an equal pressure occurs at the barrel wall and the screw base. This means that $k_{1}=k_{3}$. On the other hand, the pressure at the screw flights must be considered differently, since it changes due to the large channel width compared to the depth. A measured pressure anisotropy coefficient of $k_{2}=0.4$ is given in [3]. For the determination of the friction forces on the barrel, an averaged barrel friction value is to be used, since the barrel is interrupted in the area of the grooves by these and the internal friction value of the pellet acts there. Depending on the groove width and number, the average barrel friction coefficient is obtained as follows:

(A1) $${\bar{\mu }}_{B}=\mu_{B}+\left(\mu_{i}-\mu_{B}\right)\cdot \frac{N_{G}\cdot b_{G}}{\pi \cdot D_{B}\cdot \sin\left(\varphi_{G}\right)}$$

By setting up a force balance in the conveying direction and a moment balance in the peripheral direction, and converting this system of equations, the channel conveying angle can be calculated:

(A2) $$\tan\left(\alpha_{Ch}\right)=\frac{\sqrt{1+K^{2}-M_{1}^{2}}-K\cdot M_{1}}{K\cdot \sqrt{1+K^{2}-M_{1}^{2}}+M_{1}}$$

with

(A3) $$K=E\cdot \tan\left(\bar{\varphi }\right);\;E=1-\frac{h_{e}}{D_{B}}$$

(A4) $$\begin{array}{c} M_{1}=2\cdot \frac{k_{2}}{k_{1}}\cdot \frac{\mu_{S}}{{\bar{\mu }}_{B}}\cdot \frac{h_{e}\cdot i\cdot E}{t-\frac{e}{\cos\left(\bar{\varphi }\right)}\cdot i}\cdot \left[K\cdot \tan\left(\bar{\varphi }\right)+E\right]+\frac{k_{3}}{k_{1}}\cdot \frac{\mu_{S}}{{\bar{\mu }}_{B}}\cdot C\cdot \cos\left(\varphi_{S}\right)\\ \cdot [K\cdot \tan\left(\varphi_{S}\right)+C] \end{array}$$

(A5) $$C=1-2\cdot \frac{h_{e}}{D_{B}}$$

(A6) $$\varphi_{S}=arctan\left(\frac{t}{\pi \cdot (D_{B}-2\cdot h_{e}}\right)$$

(A7) $$\bar{\varphi }=arctan\left(\frac{t}{\pi \cdot (D_{B}-h_{e}}\right)$$

## Appendix C

**Table A3:** Design of Experiments for Numerical Simulation.

	Factor 1	Factor 2	Factor 3	Factor 4	Factor 5	Factor 6	Factor 7	Factor 8
Meaning	Internal Coefficient of Friction	Particle Diameter	Barrel Diameter	Channel Depth	Screw Pitch	Grooves Angle	Peripheral Speed	Back-Pressure
Symbol	$\mu_{i}$	$d_{p}$	$D_{B}$	$h_{e}/D_{B}$	$t/D_{B}$	$\varphi_{G}$	$v_{0}$	p
Unit	(-)	(mm)	(mm)	(-)	(-)	(°)	(m/s)	(bar)
1	0.44	1.61	45	0.12	0.82	60	0.65	401
2	0.71	1.61	45	0.12	0.82	60	0.25	109
3	0.44	3.39	45	0.12	0.82	60	0.25	109
4	0.71	3.39	45	0.12	0.82	60	0.65	401
5	0.44	1.61	75	0.12	0.82	60	0.25	401
6	0.71	1.61	75	0.12	0.82	60	0.65	109
7	0.44	3.39	75	0.12	0.82	60	0.65	109
8	0.71	3.39	75	0.12	0.82	60	0.25	401
9	0.44	1.61	45	0.18	0.82	60	0.25	401
10	0.71	1.61	45	0.18	0.82	60	0.65	109
11	0.44	3.39	45	0.18	0.82	60	0.65	109
12	0.71	3.39	45	0.18	0.82	60	0.25	401
13	0.44	1.61	75	0.18	0.82	60	0.65	401
14	0.71	1.61	75	0.18	0.82	60	0.25	109
15	0.44	3.39	75	0.18	0.82	60	0.25	109
16	0.71	3.39	75	0.18	0.82	60	0.65	401
17	0.44	1.61	45	0.12	1.18	60	0.65	109
18	0.71	1.61	45	0.12	1.18	60	0.25	401
19	0.44	3.39	45	0.12	1.18	60	0.25	401
20	0.71	3.39	45	0.12	1.18	60	0.65	109
21	0.44	1.61	75	0.12	1.18	60	0.25	109
22	0.71	1.61	75	0.12	1.18	60	0.65	401
23	0.44	3.39	75	0.12	1.18	60	0.65	401
24	0.71	3.39	75	0.12	1.18	60	0.25	109
25	0.44	1.61	45	0.18	1.18	60	0.25	109
26	0.71	1.61	45	0.18	1.18	60	0.65	401
27	0.44	3.39	45	0.18	1.18	60	0.65	401
28	0.71	3.39	45	0.18	1.18	60	0.25	109
29	0.44	1.61	75	0.18	1.18	60	0.65	109
30	0.71	1.61	75	0.18	1.18	60	0.25	401
31	0.44	3.39	75	0.18	1.18	60	0.25	401
32	0.71	3.39	75	0.18	1.18	60	0.65	109
33	0.44	1.61	45	0.12	0.82	90	0.65	109
34	0.71	1.61	45	0.12	0.82	90	0.25	401
35	0.44	3.39	45	0.12	0.82	90	0.25	401
36	0.71	3.39	45	0.12	0.82	90	0.65	109
37	0.44	1.61	75	0.12	0.82	90	0.25	109
38	0.71	1.61	75	0.12	0.82	90	0.65	401
39	0.44	3.39	75	0.12	0.82	90	0.65	401
40	0.71	3.39	75	0.12	0.82	90	0.25	109
41	0.44	1.61	45	0.18	0.82	90	0.25	109
42	0.71	1.61	45	0.18	0.82	90	0.65	401
43	0.44	3.39	45	0.18	0.82	90	0.65	401
44	0.71	3.39	45	0.18	0.82	90	0.25	109
45	0.44	1.61	75	0.18	0.82	90	0.65	109
46	0.71	1.61	75	0.18	0.82	90	0.25	401
47	0.44	3.39	75	0.18	0.82	90	0.25	401
48	0.71	3.39	75	0.18	0.82	90	0.65	109
49	0.44	1.61	45	0.12	1.18	90	0.65	401
50	0.71	1.61	45	0.12	1.18	90	0.25	109
51	0.44	3.39	45	0.12	1.18	90	0.25	109
52	0.71	3.39	45	0.12	1.18	90	0.65	401
53	0.44	1.61	75	0.12	1.18	90	0.25	401
54	0.71	1.61	75	0.12	1.18	90	0.65	109
55	0.44	3.39	75	0.12	1.18	90	0.65	109
56	0.71	3.39	75	0.12	1.18	90	0.25	401
57	0.44	1.61	45	0.18	1.18	90	0.25	401
58	0.71	1.61	45	0.18	1.18	90	0.65	109
59	0.44	3.39	45	0.18	1.18	90	0.65	109
60	0.71	3.39	45	0.18	1.18	90	0.25	401
61	0.44	1.61	75	0.18	1.18	90	0.65	401
62	0.71	1.61	75	0.18	1.18	90	0.25	109
63	0.44	3.39	75	0.18	1.18	90	0.25	109
64	0.71	3.39	75	0.18	1.18	90	0.65	401
65	0.35	2.5	60	0.15	1	75	0.45	255
66	0.8	2.5	60	0.15	1	75	0.45	255
67	0.58	1	60	0.15	1	75	0.45	255
68	0.58	4	60	0.15	1	75	0.45	255
69	0.58	2.5	35	0.15	1	75	0.45	255
70	0.58	2.5	85	0.15	1	75	0.45	255
71	0.58	2.5	60	0.1	1	75	0.45	255
72	0.58	2.5	60	0.2	1	75	0.45	255
73	0.58	2.5	60	0.15	0.7	75	0.45	255
74	0.58	2.5	60	0.15	1.3	75	0.45	255
75	0.58	2.5	60	0.15	1	50	0.45	255
76	0.58	2.5	60	0.15	1	100	0.45	255
77	0.58	2.5	60	0.15	1	75	0.11	255
78	0.58	2.5	60	0.15	1	75	0.79	255
79	0.58	2.5	60	0.15	1	75	0.45	10
80	0.58	2.5	60	0.15	1	75	0.45	500
81	0.58	2.5	60	0.15	1	75	0.45	255
82	0.58	2.5	60	0.15	1	75	0.45	255
83	0.58	2.5	60	0.15	1	75	0.45	255
84	0.58	2.5	60	0.15	1	75	0.45	255
85	0.58	2.5	60	0.15	1	75	0.45	255
86	0.58	2.5	60	0.15	1	75	0.45	255
87	0.58	2.5	60	0.15	1	75	0.45	255
88	0.58	2.5	60	0.15	1	75	0.45	255
89	0.58	2.5	60	0.15	1	75	0.45	255
90	0.58	2.5	60	0.15	1	75	0.45	255

## Appendix D. Data Used for Analytical Throughput Calculation of Validation

**Table A4:** Material.

	Factor 1	Factor 2	Factor 3	Factor 4	Factor 5
Meaning	Screw Friction Coefficient	Barrel Friction Coefficient	Internal Friction Coefficient	Standard Bulk Density	Average Pellet Diameter
Symbol	$\mu_{s}$	$\mu_{b}$	$\mu_{i}$	$\rho_{b,0}$	$d_{p}$
Unit	(-)	(mm)	(mm)	(-)	(-)
PP	0.112	0.28	0.5	540	4.55
PA6	0.068	0.17	0.37	718.6	2.68
LLDPE	0.08	0.2	0.5	550	1.41
PS small	0.156	0.39	0.38	580	0.9921
PS medium	0.156	0.39	0.38	580	1.3622
PS large	0.156	0.39	0.38	580	2.8614
